# Excitation-Controlled Host–Guest Multicolor Luminescence in Lanthanide-Doped Calcium Zirconate for Information Encryption

**DOI:** 10.3390/molecules28227623

**Published:** 2023-11-16

**Authors:** Yangbo Wang, Yingdong Han, Runfa Liu, Cunping Duan, Huaiyong Li

**Affiliations:** 1 School of Materials Science and Engineering, Liaocheng University, Liaocheng 252059, China; 2 College of Science, Civil Aviation University of China, Tianjin 300300, China; hansuo@126.com

**Keywords:** luminescence, lanthanide, multicolor emission, luminescence regulation, information encryption

## Abstract

Efficient control over lanthanide luminescence by regulating excitations offers a real-time and reversible luminescence-managing strategy, which is of great importance and highly desirable for various applications, including multicolor display and information encryption. Herein, we studied the crystal structure, luminescence properties, and mechanisms of undoped and Tb^3+^/Eu^3+^-doped CaZrO_3_ in detail. The intrinsic purple-blue luminescence from host CaZrO_3_ and the introduced green/red luminescence from guest dopants Tb^3+^/Eu^3+^ were found to have different excitation mechanisms and, therefore, different excitation wavelength ranges. This enables the regulation of luminescent color through controlling the excitation wavelengths of Tb^3+^/Eu^3+^-doped CaZrO_3_. Furthermore, preliminary applications for information encryption with these materials were demonstrated using portable UV lamps of 254 and 302 nm. This study not only promotes the development of multicolor luminescence regulation in fixed-composition materials, but also advances the practical applications of lanthanide luminescent materials in visually readable, high-level anti-counterfeiting and information encryption.

## 1. Introduction

Luminescent materials that emit various colors of light under different excitation conditions provide a simple and common means of multicolor display, imaging, security, and information encryption [1,2]. Among the various luminescent materials, lanthanide-based materials are one of the most attractive classes for researchers aiming to acquire excitation-dependent luminescence due to their unique and extensive advantages, such as rich and tunable emission bands (covering the near-infrared, visible and ultraviolet regions), narrow excitation and emission bands (for the major 4f–4f transitions), large Stokes or anti-Stokes shift, and high photostability [3,4].

A widely used strategy is integrating downconversion luminescence with upconversion luminescence. For example, NaGdF_4_:Yb,Tm@NaYF_4_:Tb@EuSe nanocomposites emit blue and purple light under 365 nm UV and 980 nm NIR irradiation, respectively [5]; NaYF_4_:Yb/Er@NaTbF_4_:Eu microcrystals show green and red light under 980 nm laser and 365 nm UV excitation, respectively [6]. Many similar systems, such as lanthanide-doped NaLuF_4_/Y_2_O_3_ composites [7], NaGdF_4_:Yb/Tm@NaGdF_4_:Ce/Mn@NaYF_4_ nanoparticles [8], CaSc_2_O_4_:Yb/Tb phosphors [9], and LiYbF_4_:Y@LiGdF_4_:Yb/Tm@LiYF_4_:Eu nanoparticles [10], also generate principally similar upconversion/downconversion emissions by altering excitation wavelengths. Furthermore, designing orthogonal upconversion processes in multilayer nanocrystals [11,12,13,14] or nanoclusters [15] can also provide varied colors of luminescence under different excitation wavelengths. For example, NaYF_4_:Yb/Tm@NaYF_4_@NaYF_4_:Er/Ho@NaYF_4_ nanocrystals have blue and yellow emissions upon 980 and 1532 nm laser excitation, respectively [16], and LiREF_4_-based core/sextuple-shell Yb,Tm/Gd/Yb,Er/Nd/Gd/Er,Tm/Gd nanocrystals emit blue, green, and red light under 980, 800, and 1532 nm excitation, respectively [12]. Although incorporating excitation-dependent upconversion has proven to be a robust method, it is obvious that the elaborate construction of a material structure is often necessary in order to prevent cross-relaxation-induced loss of excitation energy, and that excitation lasers of limited wavelengths, such as 808, 980, and 1532 nm, can be selected for upconversion luminescence. In addition, in upconversion-only luminescent systems, modulating the pulse duration or repetition frequency of the excitation laser can tailor the luminescent color efficiently and almost continuously [17,18], but this method undoubtedly relies on a complex excitation light source system.

Another major class of current luminescent materials possessing excitation-dependent luminescence usually incorporates different luminous ions with different excitation wavelengths [19], such as by co-doping Er^3+^ (shows green emission under 360–380 nm excitations) and Eu^2+^ (shows red emission under 250–320 nm excitations) in La_4_GeO_8_ [20], co-doping Er^3+^ (shows green emission under 362–380 nm excitations) and Pr^3+^ (shows red emission under 200–400 nm excitations) in NaNbO_3_ and Ca_2_Nb_2_O_7_ [21,22], and co-doping Tb^3+^ and Eu^3+^ for a tunable color from yellow to pink when increasing the excitation wavelength from 254 to 365 nm [23]. This type of luminescence modulation, independent of upconversion, supports a continuous color control when applying a continuous change in excitation wavelengths. Except for the management of luminous ions, the luminescence of host materials may offer new opportunities to achieve excitation-dependent luminescence. However, this has not attracted much attention to date, and there are very few examples: Cs_3_TbCl_6_ nanocrystals produce blue luminescence of the host under 365 nm excitation, while the green luminescence of Tb^3+^ is produced under 254 nm excitation, enabling colorful luminescence from green to blue under 260–360 nm excitations [24]. KLu_3_F_10_:Tb crystals emit blue light from defects in KLu_3_F_10_ under 365 nm excitation and green light from Tb^3+^ under 254 nm excitation, with the luminescence tuning from green to blue under 250–370 nm excitations [25]. Combining the self-trapped exciton luminescence of the matrix and the luminescence of doped lanthanide ions, Cs_2_Ag_0.3_Na_0.7_InCl_6_:Yb^3+^/Eu^3+^/Ho^3+^ microcrystals display yellow, red, and green light under 300, 394, and 980 nm excitations, respectively [26], and a similar phenomenon has been found in ZrO_2_:Gd^3+^ nanoparticles [27]. It is obvious that more effort is needed in order to explore host-luminescence-assisted robust luminescence modulation under varied excitation wavelengths.

Previous reports have shown that CaZrO_3_, one ABO_3_-type perovskite oxide, can emit an intense purple-blue light with a broad emission band ranging from about 350 to 550 nm under excitations of 200–300 nm, where the excitation is derived from the host absorption and the emission derives from the oxygen-defect-related radiative transitions [28,29]. Importantly, the distorted perovskite structure of CaZrO_3_ provides two kinds of lattice sites with different symmetries, eight-fold-coordinated Ca^2+^ sites and six-fold-coordinated Zr^4+^ sites to accommodate various trivalent lanthanide ions (Ln^3+^) for multimode and multicolor luminescence [30,31,32,33,34,35]. Great progress has been made in the research on doping chemistry and luminescence properties in CaZrO_3_ in the last decade or so. For example, Kunti et al. investigated the structural and luminescence properties of CaZrO_3_:Eu^3+^ phosphors and disclosed the role of oxygen vacancy in the origin of the host CaZrO_3_ emissions and the energy transfer mechanism through detailed experimental and theoretical research [36]. Zhang et al. achieved tunable, full-color luminescence by managing the composition and doping concentration in Tb^3+^/Eu^3+^-doped CaZrO_3_ phosphors [37]. Very recently, Ueda et al. revealed that doped Eu^3+^ ions occupy not only A sites, but also B sites, in CaZrO_3_, and that co-doping ions of different sizes can regulate the site-occupation proportions as well as the site-dependent Eu^3+^ luminescence [38]. However, it is clear that adjusting the dopant concentration [36,39] and introducing ions of different luminous colors, including different Ln^3+^ ions [37,40] and non-rare-earth ions [41,42], are still the principal strategies by which to achieve multicolor luminescence; therefore, multicolor luminescence is still absent in composition-fixed Ln^3+^-doped CaZrO_3_. In addition, for the recently studied Tb^3+^/Eu^3+^ co-doped colorful phosphors beyond CaZrO_3_, such as La_4_GeO_8_:Tb/Eu [43], K_5_Eu(MoO_4_)_4_:Tb [44], MgF_2_:Tb/Eu [45], K_3_Lu(PO_4_)_2_:Tb/Eu [46], Sr_3_MgSi_2_O_8_:Eu/Tb [47], and Gd_2_B_2_WO_9_:Eu/Tb [48], excitation-dependent multicolor luminescence is also very rare.

In this work, we achieved multicolor luminescence in Tb^3+^/Eu^3+^-doped CaZrO_3_ by managing not only the luminous ions, but also the excitation wavelengths. We introduced green and red luminescence in purple-blue-emitting CaZrO_3_ polycrystalline powders by doping Tb^3+^ and Eu^3+^, respectively. We investigated the doping chemistry of Tb^3+^/Eu^3+^ in CaZrO_3_ and the luminescence properties, as well as the mechanism. The results revealed that the luminescence from the host CaZrO_3_ and from guest dopants Tb^3+^ and Eu^3+^ go through completely different energy paths and require excitation light in different wavelength regions. This allows us to control the luminescent color of these materials by succinctly modulating excitation wavelengths. In addition, we demonstrated a group of prototypes to be utilized for anti-counterfeiting and information encryption using undoped and Tb^3+^/Eu^3+^-doped CaZrO_3_.

## 2. Results and Discussion

### 2.1. Structural and Compositional Analysis of Undoped and Tb^3+^/Eu^3+^-Doped CaZrO_3_

**Figure 1:**
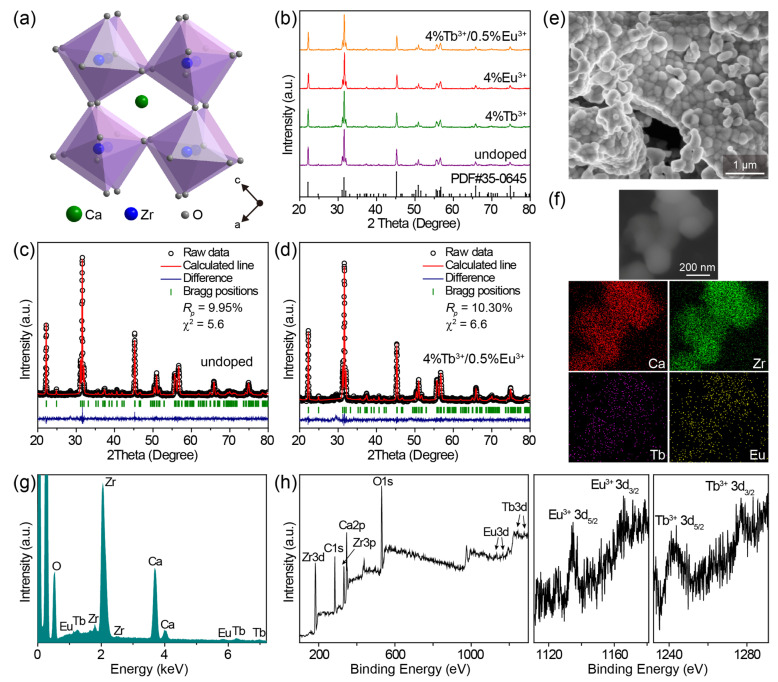
(**a**) Crystal structure diagram of CaZrO_3_. (**b**) Powder XRD patterns of representative undoped CaZrO_3_, CaZrO_3_:4%Tb^3+^, CaZrO_3_:4%Eu^3+^, and CaZrO_3_:4%Tb^3+^/0.5%Eu^3+^. The bars at the bottom are the reference standard patterns of CaZrO_3_ (PDF card No. 35–0645). Rietveld refinement results of (**c**) undoped CaZrO_3_ and (**d**) CaZrO_3_:4%Tb^3+^/0.5%Eu^3+^. (**e**) SEM image and (**f**) elemental mapping of CaZrO_3_:4%Tb^3+^/0.5%Eu^3+^. (**g**) EDS spectrum of CaZrO_3_:4%Tb^3+^/0.5%Eu^3+^. (**h**) XPS spectra of CaZrO_3_:4%Tb^3+^/0.5%Eu^3+^: the overall spectrum, high resolution spectra of Eu 3d and Tb 3d.

The CaZrO_3_ crystal adopted a distorted perovskite GdFeO_3_-type structure (Figure 1a), wherein the array of ZrO_6_ octahedra constituted the crystal structure network and Ca^2+^ ions were interspersed among ZrO_6_ octahedra [41,49]. The rotation of the ZrO_6_ octahedra led to a decrease in the coordination number (CN) of Ca^2+^ from 12 to 8 [39]; thus, the dopants Tb^3+^/Eu^3+^ would theoretically prefer to be located at the asymmetric 8-fold-coordinated Ca^2+^ sites because of a lower degree of mismatch in effective ionic radii (1.18/1.206 Å of Tb^3+^/Eu^3+^ and 1.26 Å of Ca^2+^ when CN = 8) compared with the symmetric 6-fold-coordinated Zr^4+^ sites (1.063/1.087 Å of Tb^3+^/Eu^3+^ and 0.86 Å of Zr^4+^ when CN = 6) [50].

Powder X-ray diffraction (XRD) patterns of as-synthesized pristine CaZrO_3_, CaZrO_3_:Tb^3+^, CaZrO_3_:Eu^3+^, and CaZrO_3_:Tb^3+^/Eu^3+^ powders are shown in Figure 1b and Figure S1a, and all the diffraction peaks of samples with Tb^3+^/Eu^3+^ concentrations lower than 6% can be well-indexed to the standard diffraction peaks of orthorhombic CaZrO_3_ (PDF card No. 35–0645), suggesting the successful integration of Tb^3+^/Eu^3+^ into the CaZrO_3_ matrix. A close observation of the XRD patterns revealed a few negligible impurity peaks at 28.5°–30.2° when the Tb^3+^/Eu^3+^ concentrations were larger than 6%, which was most likely due to the formation of trace amounts of Tb_2_O_3_/Eu_2_O_3_ impurities. The diffraction peak positions of the (202) planes slightly shifted toward the large-angle side after Tb^3+^/Eu^3+^ doping (Figure S1b), implying a shrinking of the crystal lattice owing to the substitution of big Ca^2+^ by small Tb^3+^/Eu^3+^. A reverse shift to the small-angle side was also observed at high Tb^3+^/Eu^3+^ doping concentrations, which suggests that Tb^3+^/Eu^3+^ ions occupy not only Ca^2+^ sites, but also Zr^4+^ sites. Partial substitution for Zr^4+^ sites is reasoned as a self-compensation for the charge imbalance caused by substituting Ca^2+^ sites, being described as: ${\mathrm{Ln}}_{\mathrm{Ca}}^{•}$ + ${\mathrm{Ln}}_{\mathrm{Zr}}^{’}$ (Ln = Tb, Eu). This has been definitively verified in recent research [38], and will also be reflected in the latter spectra of CaZrO_3_:Eu^3+^.

We conducted Rietveld refinement to evaluate the change in crystal structure induced by Tb^3+^/Eu^3+^ doping. Relevant results for undoped CaZrO_3_ and representative CaZrO_3_:4%Tb^3+^/0.5%Eu^3+^ are given in Figure 1c,d and Table S2. Based on the reliable refinement and the good agreement between the calculated and measured patterns, both samples possessed pure orthorhombic structures with the *Pnma* space group. It is worth noting that the values of lattice cell parameters, including *a*, *b*, *c*, and *V*, showed only an extremely negligible reduction after Tb^3+^/Eu^3+^ doping. For example, the cell volume *V* shrank very slightly, from 258.39 to 258.08 Å^3^. This result further confirms the speculation that a fair fraction of Tb^3+^/Eu^3+^ ions substitute smaller Zr^4+^ ions to offset the lattice shrinkage caused by substituting bigger Ca^2+^ ions. In the meanwhile, the deviation of lattice cell parameters between doped and undoped samples was less than 1%, implying no second-phase emergence and the successful doping of Tb^3+^/Eu^3+^. The slightly high *Rp* values for both undoped and Tb^3+^/Eu^3+^-doped samples may have been due to the relatively strong background signal in the XRD patterns, which was likely due to the slightly low crystallinity. However, due to the consistent measurement conditions as well as the refinement results for both the undoped and doped samples, it probably had no effect on the comparison of their crystal structure data.

The representative SEM image of CaZrO_3_:4%Tb^3+^/0.5%Eu^3+^ shows agglomerates of irregular nanoparticles with sizes about 150–350 nm (Figure 1e), which may indicate slightly low crystallinity of the obtained samples. The two-dimensional elemental mapping results in Figure 1f indicate the existence of Tb and Eu elements in the CaZrO_3_ crystals, which was also proven by the clearly identified peaks from Zr, Ca, Tb, and Eu in the EDS spectrum in Figure 1g. This EDS analysis further verified the successful doping of Tb^3+^/Eu^3+^ ions in CaZrO_3_. XPS spectra of the representative CaZrO_3_:4%Tb^3+^/0.5%Eu^3+^ sample show the peaks of not only the Ca, Zr and O elements from the host, but also Tb and Eu elements from the dopants (Figure 1h). Two peaks, around 1242.1 and 1277.0 eV, were the typical XPS peaks of Tb^3+^ 3d_5/2_ and Tb^3+^ 3d_3/2_, and two around 1134.7 and 1164.2 eV were the characteristic peaks of Eu^3+^ 3d_5/2_ and Eu^3+^ 3d_3/2_ [51], respectively, suggesting the successful introduction of Tb^3+^/Eu^3+^ into CaZrO_3_.

### 2.2. Luminescent Properties and Mechanisms of Undoped CaZrO_3_, CaZrO_3_:Tb^3+^, and CaZrO_3_:Eu^3+^

**Figure 2:**
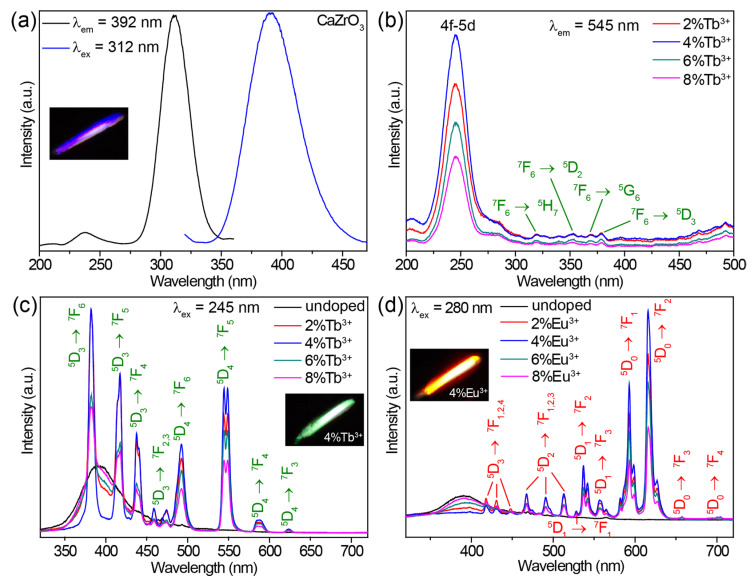
(**a**) Excitation and emission spectra of undoped CaZrO_3_. (**b**) Excitation spectra of CaZrO_3_:Tb^3+^ (2–8%), obtained by monitoring the emission at 545 nm. (**c**) Emission spectra of CaZrO_3_:Tb^3+^ (0–8%) under 245 nm excitation. (**d**) Emission spectra of CaZrO_3_:Eu^3+^ (0–8%) under 280 nm excitation. The insets in (**a**,**c**,**d**) are photographs of the corresponding samples.

The undoped CaZrO_3_ sample exhibited a broad emission band centered at 392 nm under 312 nm excitation (Figure 2a). A bright purple-blue luminescence is shown in the inset of Figure 2a. When monitoring the 392 nm emission, an excitation spectrum comprising a strong band of around 312 nm, along with a weak band of around 237 nm, was obtained (Figure 2a). The excitation and emission mechanisms of undoped CaZrO_3_ have been thoroughly explored, both theoretically and experimentally, in previous research. The weak excitation band at 237 nm (5.23 eV) can be attributed to the absorption of CaZrO_3_ (that is, the band-gap transition) as a result of O^2−^ → Zr^4+^ electron transfer in ZrO_6_ octahedra, according to previous reports [28,29]. The similar value of the band gap energy (5.55 eV, 223 nm) obtained from the absorption spectrum of CaZrO_3_ verified this definitively (Figure S2). The intense excitation band around 312 nm may have been due to the absorption of oxygen vacancies, which generated defect states in the bandgap of CaZrO_3_ [52]. These oxygen vacancies were a singly ionized oxygen vacancy ($V_{O}^{•}$) and doubly ionized one ($V_{O}^{••}$), and occurred in the form of complex [CaO_7_·$V_{O}^{•}$], [CaO_7_·$V_{O}^{••}$], [ZrO_5_·$V_{O}^{•}$], and [ZrO_5_·$V_{O}^{••}$] clusters generated during the high temperature synthesis processes [29,36]. The emission band stemmed from the radiative transitions of deep oxygen vacancy states. 

Doping Tb^3+^ or Eu^3+^ ions into CaZrO_3_ changed the luminescent properties significantly. The excitation spectra of CaZrO_3_:Tb^3+^ (2–8%) monitoring Tb^3+^ emission at 545 nm are presented in Figure 2b. All excitation spectra showed strong and broad excitation bands centered at 245 nm and several weak and narrow excitation peaks in the range of 310–380 nm. These weak and narrow excitation peaks matched a series of Tb^3+^ 4f–4f transitions well. The intense and broad excitation band could be attributed to the characteristic absorption from the 4f–5d transitions of Tb^3+^ [53,54]. The completely different excitation band position around 245 nm compared with the main excitation band around 312 nm of undoped CaZrO_3_ could exclude the host’s absorption of CaZrO_3_. This was further confirmed by the emission spectra under 312 nm excitation (Figure S3a). Obviously, the intense excitation at 312 nm of undoped CaZrO_3_ was unable to trigger the luminescence of Tb^3+^ at all. Furthermore, the almost identical excitation band positions at 245 nm for samples with varied Tb^3+^ concentrations also opposed the possible Tb^3+^-doping-induced shift of the host absorption band. Figure 2c compares the emission spectra of CaZrO_3_:Tb^3+^ (0–8%) under 245 nm excitation, showing that CaZrO_3_:Tb^3+^ (2–8%) presented a series of strong and narrow emission peaks originating from the characteristic 4f–4f transitions of Tb^3+^. The broad emission bands around 392 nm from the host CaZrO_3_ still appeared, although the intensity was reduced. This is because the excitation wavelength of 245 nm overlapped with the tail of the excitation band for host emission. The Tb^3+^ luminescence reached the highest intensity in CaZrO_3_:4%Tb^3+^, and concentrations greater than 4% led to a decline in intensity due to concentration quenching. Digital photographs display the bright green luminescence of CaZrO_3_:Tb^3+^ (2–8%) (the inset in Figure 2c and Figure S3b).

The excitation spectra of CaZrO_3_:Eu^3+^ (2–8%) in Figure S4a show a broad band around 280 nm and several narrow peaks. These narrow excitation peaks are all from the 4f–4f transitions of Eu^3+^ as marked. The broad excitation band around 280 nm is ascribed to the charge transfer (CT) transitions from O 2p states to Eu 4f states [38,39,55]. Similarly, the significant difference (about 32 nm) in the excitation band positions between CaZrO_3_:Eu^3+^ and undoped CaZrO_3_, together with the almost identical excitation band positions at 280 nm for samples with varied Eu^3+^ concentrations, also rule out the possibility of host absorption from CaZrO_3_. When excited by 280 nm UV light, CaZrO_3_:Eu^3+^ (2–8%) samples showed a series of sharp emissions from Eu^3+^ 4f–4f transitions in the visible region, as well as a weak and wide emission band around 392 nm from the host CaZrO_3_ (Figure 2d). The obviously stronger emissions at 593 and 616 nm made the overall luminescence bright red for CaZrO_3_:Eu^3+^ (the inset in Figure 2d and Figure S4b). As is known, the local symmetry of Eu^3+^ ions determines the relative intensity between the 616 nm emission from ^5^D_0_ → ^7^F_2_ electric dipole transitions and the 593 nm emission from ^5^D_0_ → ^7^F_1_ magnetic dipole transitions [56]. The emission at 616 nm dominated in the emission spectrum for Eu^3+^ ions in asymmetric lattice sites, while the 593 nm emission was dominant for Eu^3+^ ions in symmetric sites. Herein, the slightly weaker emission intensity at 593 nm compared with 616 nm revealed that a fair amount of Eu^3+^ ions were located at the symmetric centers of the ZrO_6_ octahedra. This is consistent with the negligible changes in crystal structure parameters after doping Tb^3+^/Eu^3+^. A concentration quenching causing a drop in luminescence intensity was also observed when the Eu^3+^ concentration was greater than 4%. In addition, Eu^3+^ ions could also be efficiently excited by their own 4f–4f transitions, but we did not focus on this in the present research because the sharp excitation peak did not lead to a continuous change in luminescence color when tuning excitation wavelengths.

We explored the mechanism of the concentration-quenching phenomenon in CaZrO_3_:Tb^3+^ and CaZrO_3_:Eu^3+^. As is well known to us, increasing the doping concentration of activators causes decreased interionic distance and promotes non-radiative energy transfer among activators [57]. We first estimated the critical distance (*R_c_*) among Tb^3+^/Eu^3+^ ions in CaZrO_3_ using the Blasse formula [58]:

(1) $${R_{c}=2(\frac{3V}{4\pi X_{c}Z})}^{1/3}$$

where *V* is the unit cell volume (258.3 Å^3^ for CaZrO_3_), *X_c_* is the critical doping concentration (0.04 for Tb^3+^/Eu^3+^ herein), and *Z* is the number of lattice sites available for Tb^3+^ or Eu^3+^ occupation per unit cell (*Z* = 4 for CaZrO_3_). The calculated *R_c_* was about 14.56 Å; thus, the exchange interaction could be excluded from the possible mechanism of concentration quenching because it mainly occurred when *R_c_* was less than 5 Å. Therefore, we reasoned that the multipole–multipole interaction was principally responsible for the concentration quenching. We further determined the mode of multipole–multipole interaction to be dipole–dipole interaction (find details in the Supporting Information and Figure S5). Therefore, the concentration quenching in CaZrO_3_:Tb^3+^ and CaZrO_3_:Eu^3+^ can be attributed to electric dipole–dipole interaction.

**Figure 3:**
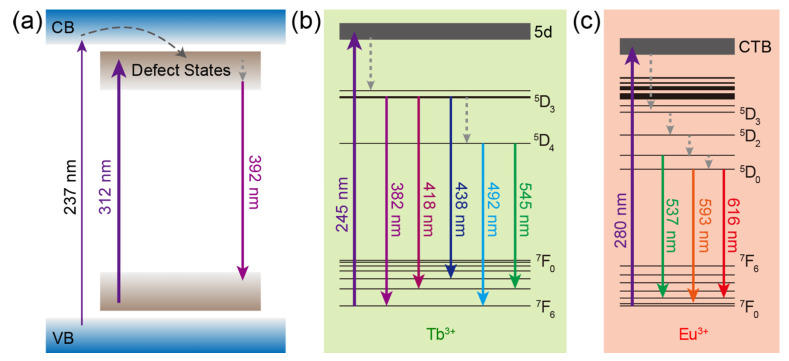
Schematics of the main luminescence mechanism in (**a**) CaZrO_3_, (**b**) CaZrO_3_:Tb^3+^, and (**c**) CaZrO_3_:Eu^3+^. Some of the energy levels of Tb^3+^ and Eu^3+^ are shown and marked. The upward/downward full arrows represent the excitation/emission processes. The curved and straight dash arrows represent the energy transfer and non-radiative transition processes, respectively. CTB: charge transfer band.

The mechanisms of luminescence from CaZrO_3_, CaZrO_3_:Tb^3+^, and CaZrO_3_:Eu^3+^ are summarized and depicted in Figure 3. For CaZrO_3_, the excitation of electrons from the ground levels to the excited levels of defect states, the subsequent non-radiative transitions to deep defect states, and the following radiative recombinations were the main energy paths, meanwhile weak excitation could also be accessed by the band-gap transitions and the subsequent energy transfer to the defect states (Figure 3a). In CaZrO_3_:Tb^3+^, the main mechanism for Tb^3+^ luminescence was the self-excitation of Tb^3+^ through 4f–5d transitions followed by the radiative 4f–4f transitions, accompanied by some non-radiative transitions (Figure 3b). As for CaZrO_3_:Eu^3+^, the main luminescent mechanism was the excitation by the O^2−^ → Eu^3+^ charge transfer transitions, which led to the described radiative 4f–4f transitions of Eu^3+^ (Figure 3c). It is obvious that the main excitation channels for the luminescence from host CaZrO_3_ and dopants Tb^3+^ and Eu^3+^ were completely different, as were the excitation wavelengths.

### 2.3. Luminescent Properties and Mechanisms of CaZrO_3_:Tb^3+^/Eu^3+^

**Figure 4:**
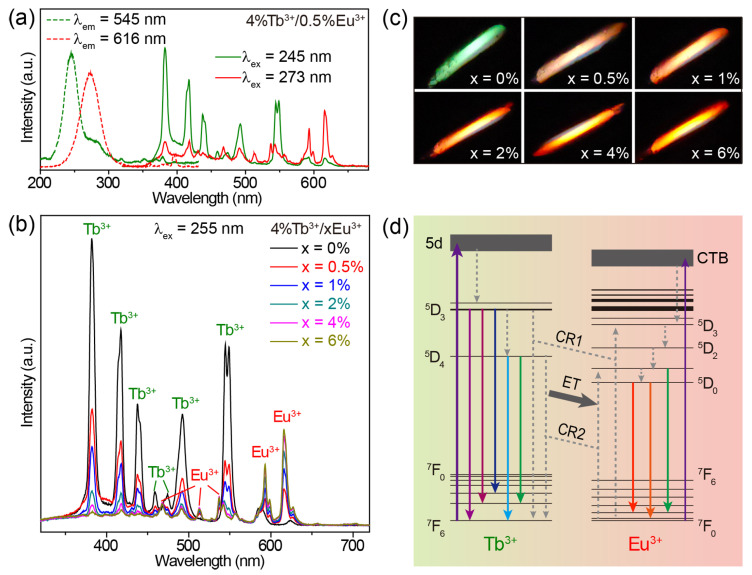
(**a**) Excitation and emission spectra of CaZrO_3_:4%Tb^3+^/0.5%Eu^3+^. (**b**) Emission spectra and (**c**) photographs of CaZrO_3_:4%Tb^3+^/xEu^3+^ (x = 0–6%) under 255 nm excitation. (**d**) Schematic of the main luminescence mechanism of CaZrO_3_:4%Tb^3+^/xEu^3+^. Some energy levels of Tb^3+^ and Eu^3+^ are shown and marked.

When co-doping Tb^3+^ and Eu^3+^ ions into CaZrO_3_, we obtained the emissions of Tb^3+^ and Eu^3+^ simultaneously in a single material. The excitation spectra of CaZrO_3_:4%Tb^3+^/0.5%Eu^3+^ monitoring the 545 nm emission of Tb^3+^ and the 616 nm emission of Eu^3+^ show two broad excitation bands centered at 245 and 273 nm, respectively (Figure 4a). These two bands were almost identical to the excitation bands in Tb^3+^-singly-doped and Eu^3+^-singly-doped CaZrO_3_; therefore, it is reasonable to allocate them to the 4f–5d transitions of Tb^3+^ and the O^2−^ → Eu^3+^ charge transfer transitions, respectively. The excitation band at 245 nm for Tb^3+^ 545 nm emissions showed no shift in peak position, but only a decrease in intensity when the concentration of Eu^3+^ was increased from 0.5% to 6% (Figure S6a), because doping Eu^3+^ induces the gradual weakening of Tb^3+^ luminescence. The increase in the excitation band, which was around 273 nm for Eu^3+^ 616 nm emissions, implies an opposite trend regarding Eu^3+^ emission intensity (Figure S6b). To obtain intensity-comparable luminescence from both Tb^3+^ and Eu^3+^, we selected 255 nm, approximately located at the intersection of the two excitation bands, as the excitation wavelength to obtain the emission spectra.

As shown in Figure 4b, when increasing the concentration of Eu^3+^ in CaZrO_3_:4%Tb^3+^/xEu^3+^ (x = 0–6%), the characteristic emissions of Eu^3+^ became gradually stronger, while the emission intensity of Tb^3+^ showed the opposite trend under the excitation of 255 nm. This fits well with the luminescent color change from green to red (Figure 4c). Additionally, the broad emission band around 392 nm in the host CaZrO_3_ maintained a low intensity in all Tb^3+^/Eu^3+^-doubly-doped CaZrO_3_ under the excitation of 255 nm. For a Tb^3+^ emission decline by adding Eu^3+^ content, the unchanged content of Tb^3+^ could exclude the possible effect of cross-relaxation (CR) processes (such as familiar CR: ^5^D_3_ + ^7^F_6_ → ^5^D_4_ + ^7^F_0,1_) between Tb^3+^ ions. Additional Eu^3+^ content could increase the CR processes between Tb^3+^ and Eu^3+^, as in Figure 4d, mainly including CR1: ^5^D_3_ (Tb^3+^) + ^7^F_J_ (Eu^3+^) → ^7^F_J_ (Tb^3+^) + ^5^D_3_ (Eu^3+^) and CR2: ^5^D_4_ (Tb^3+^) + ^7^F_J_ (Eu^3+^) → ^7^F_J_ (Tb^3+^) + ^5^D_0,1_ (Eu^3+^). These CR processes would promote the generation of Eu^3+^-emissive levels of ^5^D_0_ and ^5^D_1_ while dissipating Tb^3+^-emissive levels of ^5^D_3_ and ^5^D_4_, leading to the Tb^3+^ → Eu^3+^ energy transfer, which is widely considered to be the reason for decreasing Tb^3+^ emissions [59,60]. In addition, the Eu^3+^ emissions of CaZrO_3_:4%Tb^3+^/xEu^3+^ samples continue to enhance slightly when the Eu^3+^ concentration is 6%, which exceeds the optimal doping concentration of Eu^3+^ singly-doped samples, which is 4%. This could be attributed to possible compensation by the Tb^3+^ → Eu^3+^ energy transfer to increased concentration quenching between Eu^3+^ ions. Therefore, the main luminescent mechanism in CaZrO_3_:Tb^3+^/Eu^3+^ can be described as follows (Figure 4d): Being excited by the 4f–5d transitions, Tb^3+^ ions can not only emit light through radiative 4f–4f transitions, but also transfer partial excitation energy to Eu^3+^ through CR1 and CR2 processes. Then, excited Eu^3+^ ions emit light through radiative 4–4f transitions; Eu^3+^ ions can also be excited by O^2−^ → Eu^3+^ charge transfer transitions.

**Figure 5:**
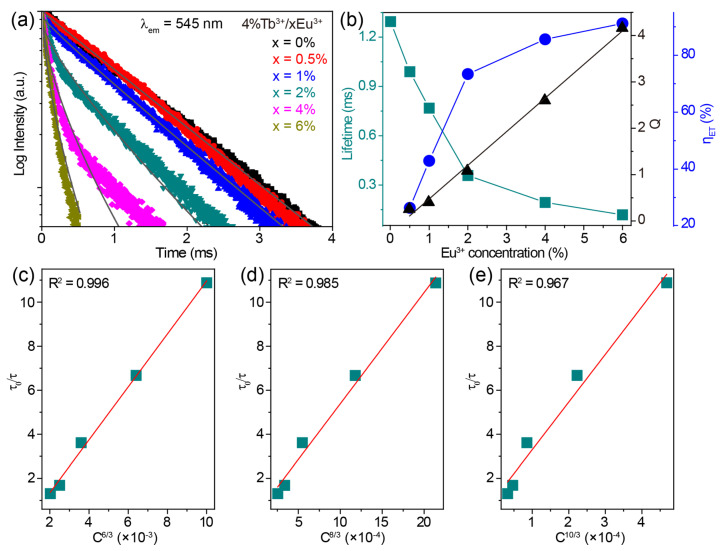
(**a**) The decay curves monitoring the 545 nm emissions of CaZrO_3_:4%Tb^3+^/xEu^3+^ (x = 0–6%) under 255 nm excitation. I-H fitting results of the corresponding samples at S = 6 are shown as grey lines. (**b**) The calculated lifetime values of Tb^3+ 5^D_4_ → ^7^F_5_ transition, energy transfer parameter Q, and efficiency (*η_ET_*) from Tb^3+^ to Eu^3+^ in CaZrO_3_:4%Tb^3+^/xEu^3+^ (x = 0–6%) as a function of Eu^3+^ concentration. The relationship of *τ_0_*/*τ* of Tb^3+^ to (**c**) *C*^6/3^, (**d**) *C*^8/3^ and (**e**) *C*^10/3^ in CaZrO_3_:4%Tb^3+^/xEu^3+^ (x = 0.5–6%).

The luminescence decay curves monitoring the 545 nm emissions of CaZrO_3_:4%Tb^3+^/xEu^3+^ (x = 0–6%) under 255 nm excitation are shown in Figure 5a. These decay curves show obvious non-exponential patterns, especially for Tb^3+^/Eu^3+^-co-doped samples, so the effective lifetime was adopted and calculated using the following equation:

(2) $$\tau_{\mathrm{eff}}=\frac{\int tI\left(t\right)dt}{\int I\left(t\right)dt}$$

where *I*(*t*) is the luminescence intensity at time *t*. The calculated values of the effective lifetimes of the Tb^3+^ 545 nm emission decreased from 1.345 to 0.119 ms as the concentration of Eu^3+^ increased from 0 to 6% in CaZrO_3_:4%Tb^3+^/xEu^3+^ (Figure 5b). This downward trend in lifetime implies the gradually reduced probability of Tb^3+ 5^D_4_ → ^7^F_5_ radiative transitions being due to the energy transfer from Tb^3+^ to Eu^3+^. The efficiency of this energy transfer (*η_ET_*) was estimated based on the following equation:

*η_ET_* = (1 − *τ/τ*_0_) × 100% (3)

where *τ*_0_ and *τ* are the lifetimes of Tb^3+^ 545 nm emissions without and with co-doped Eu^3+^, respectively. The calculated values of *η_ET_* were 26.39%, 42.90%, 73.38%, 85.58%, and 91.15% for 0.5%, 1%, 2%, 4%, and 6% Eu^3+^ in CaZrO_3_:4%Tb^3+^/xEu^3+^, respectively (Figure 5b).

To determine the energy transfer mechanism, the critical distance between Tb^3+^ and Eu^3+^ was calculated to be 14.00 Å using the Equation (1), indicating that the multipole–multipole interaction dominates the energy transfer processes. The following formula for the relationship between the lifetime and the doping concentration was used to further disclose the mode of multipole–multipole interaction [51]:

(4) $$\frac{\tau_{0}}{\tau }\propto C^{n/3}$$

where *C* is the total concentration of Tb^3+^ and Eu^3+^, and the values of *n* at 6, 8, and 10 correspond to dipole–dipole, dipole–quadrupole, and quadrupole–quadrupole interaction, respectively. The dependences of *τ*_0_/*τ* on *C^n^*^/3^ were plotted and fitted linearly, as shown in Figure 5c–e. The optimal fitting coefficient *R*^2^ was obtained at *n* = 6, suggesting that the main energy transfer mechanism was a dipole–dipole interaction.

Luminescence decay analysis according to the Inokuti–Hirayama (I-H) model also revealed the multipole–multipole interaction mechanism between Tb^3+^ and Eu^3+^ [61]. The I-H model described the luminescence decay intensity *I*(*t*) at time *t* using the following equation:

(5) $$\;I(t)\;=I_{0}\exp[-(\frac{t}{\tau_{0}})\;-\;{Q(\frac{t}{\tau_{0}})}^{3/s}]$$

where *I*_0_ is the intensity when *t* = 0; *τ*_0_ is the intrinsic lifetime of Tb^3+^ without Eu^3+^ (1.345 ms), and Q is the energy transfer parameter, defined as:

(6) $$Q=\frac{4\pi }{3}\Gamma (1\;-\;\frac{3}{s})N_{0}R_{0}^{3}$$

where *Γ* is the Euler function, *N*_0_ is the Eu^3+^ concentration, and *R*_0_ is the critical distance. The values of S at 6, 8, and 10 correspond to dipole–dipole, dipole–quadrupole, and quadrupole–quadrupole interactions, respectively. As shown in Figure 5a, the decay curves of CaZrO_3_:4%Tb^3+^/xEu^3+^ (x = 0.5–6%) samples were fitted well by Equation (5) at S = 6. The energy transfer parameter Q, achieved from the fitting process, showed an almost linear dependence on Eu^3+^ concentration (Figure 5b), as reflected by Equation (6). This result further corroborates the notion that the dipole–dipole interaction was mainly responsible for the Tb^3+^ → Eu^3+^ energy transfer.

### 2.4. Excitation Controlled Multicolor Luminescence

**Figure 6:**
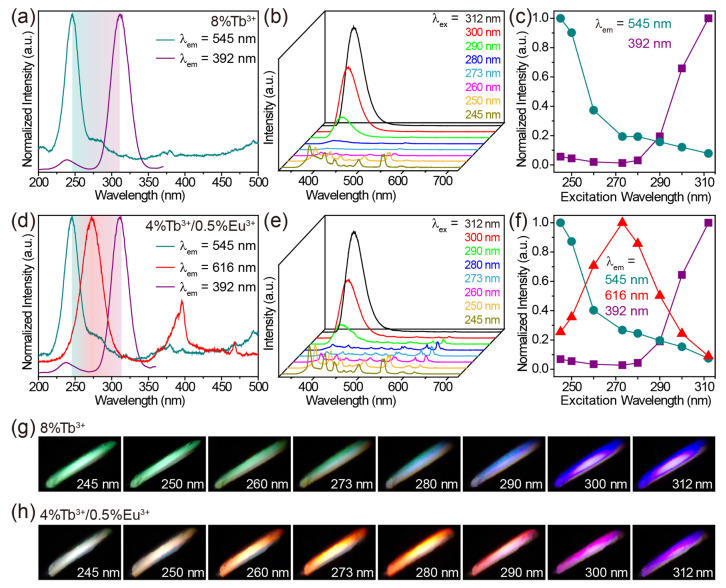
(**a**) Excitation spectra of CaZrO_3_:8%Tb^3+^, obtained by monitoring emissions at 545 and 392 nm. (**b**) Emission spectra and (**c**) intensity variation of CaZrO_3_:8%Tb^3+^ under 245–312 nm excitations. (**d**) Excitation spectra of CaZrO_3_:4%Tb^3+^/0.5%Eu^3+^, obtained by monitoring emissions at 545, 616 and 392 nm. (**e**) Emission spectra and (**f**) intensity variation of CaZrO_3_:4%Tb^3+^/0.5%Eu^3+^ under 245–312 nm excitations. Digital photographs show the color evolution of (**g**) CaZrO_3_:8%Tb^3+^ and (**h**) CaZrO_3_:4%Tb^3+^/0.5%Eu^3+^ under different excitation wavelengths.

On the basis of the above results, it is clear that the purple-blue luminescence of the host CaZrO_3_, the green luminescence of Tb^3+^ and the red luminescence of Eu^3+^ could be achieved under distinctly different excitation wavelengths in Tb^3+^/Eu^3+^-doped CaZrO_3_. This allows us to manipulate the luminescence color of these materials by simply controlling the excitation wavelengths, without the need to change the composition of the material. For Tb^3+^-singly-doped, Eu^3+^-singly-doped, and Tb^3+^/Eu^3+^-doubly-doped CaZrO_3_, we selected CaZrO_3_:8%Tb^3+^, CaZrO_3_:6%Eu^3+^, and CaZrO_3_:4%Tb^3+^/0.5%Eu^3+^, respectively, for excitation wavelength-dependent luminescence research as the strongest emissions from the host CaZrO_3_ at 392 nm in their respective groups (Figure S7). In CaZrO_3_:8%Tb^3+^, the optimal excitation wavelengths for 545 nm luminescence of Tb^3+^ and 392 nm luminescence of host CaZrO_3_ were 245 and 312 nm, respectively (Figure 6a). Thus, changing the excitation wavelength from 245 to 312 nm led to a gradual decrease in Tb^3+^ luminescence and a gradual increase in CaZrO_3_ luminescence (Figure 6b,c). The photographs show a luminescent color evolution from green to purple-blue under excitation at increased wavelengths (Figure 6g). For CaZrO_3_:6%Eu^3+^, we tuned the luminescent color from red to wine red by gradually increasing the excitation wavelength from 280 to 312 nm (Figure S8). Purple-blue emissions were not obtained due to the relatively large degree of overlap between the two excitation bands for the 616 and 392 nm emissions (Figure S8a). In CaZrO_3_:4%Tb^3+^/0.5%Eu^3+^, richer emission color variations were achieved. As shown in Figure 6d, the most effective excitation wavelengths for green emission at 545 nm, red emission at 616 nm, and purple-blue emission at 392 nm were 245, 273, and 312 nm, respectively. Therefore, when the excitation wavelengths were set to vary from 245 to 312 nm, the emissions of Tb^3+^ were gradually reduced. The emissions of host CaZrO_3_ were first slightly reduced, then significantly enhanced, while the emissions of Eu^3+^ experienced a significant rise and then a significant decline (Figure 6e,f). For the overall luminescence, the color varied from white to red, and to purple in the end (Figure 6h). A white, instead of green, luminescence was obtained under the 245 nm excitation, as it was a mixture of several intensity-comparable emissions in the range of 400–550 nm from both the CaZrO_3_ and Tb^3+^ hosts.

### 2.5. Cases of Information Encryption

**Figure 7:**
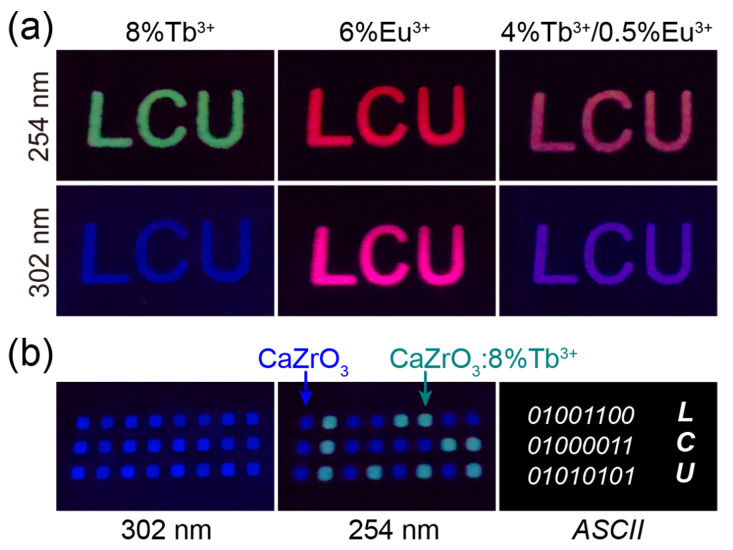
(**a**) Photographs of “LCU” patterns composed of CaZrO_3_:8%Tb^3+^, CaZrO_3_:6%Eu^3+^, and CaZrO_3_:4%Tb^3+^/0.5%Eu^3+^ under 254 and 302 nm UV light. (**b**) Photographs of array patterns composed of CaZrO_3_ and CaZrO_3_:8%Tb^3+^ under 302 and 254 nm UV light; ASCII codes revealed by the luminescent color, and cryptographic information of “LCU”.

The excitation-dependent multicolor luminescence of Tb^3+^/Eu^3+^-doped CaZrO_3_ suggests possible applications in the fields of optical anti-counterfeiting and information encryption. We established a series of luminescent patterns by selectively coating slurries of luminescent samples on PMMA plates engraved with designed patterns. As shown in Figure 7a, the “LCU” patterns, composed of CaZrO_3_:8%Tb^3+^, CaZrO_3_:6%Eu^3+^, and CaZrO_3_:4%Tb^3+^/0.5%Eu^3+^, were green, red, and light-red under 254 nm UV light, and blue, wine-red, and purple-blue under 302 nm UV light, respectively. This demonstrates the potential application of these materials in high-level anti-counterfeiting. In Figure 7b, it is shown that all signal points in a 3 × 8 dot matrix composed of both CaZrO_3_ and CaZrO_3_:8%Tb^3+^ emitted blue light under 302 nm UV light; however, the points of CaZrO_3_:8%Tb^3+^ turned to green, while the points of CaZrO_3_ remained blue under 254 nm UV light. This reveals latent optical information and can be decoded as a binary code using “1” and “0”; furthermore, the obtained 8-bit ASCII codes from the three rows of light signal can be read out as “LCU”. This preliminary experiment may provide great opportunities for optical information encryption and storage.

## 3. Materials and Methods

### 3.1. Materials and Synthesis

The reagents used in the synthesis of undoped CaZrO_3_, CaZrO_3_:Tb^3+^, CaZrO_3_:Eu^3+^, and CaZrO_3_:Tb^3+^/Eu^3+^ phosphors include CaCO_3_ (99.99%, Aladdin), ZrO_2_ (99.99%, Aladdin), Tb_2_O_3_ (99.99%, Aladdin), and Eu_2_O_3_ (99.99%, Aladdin). All reagents were used without further purification. The high temperature, solid-state reaction method was used for sample synthesis. The stoichiometric contents of CaCO_3_, ZrO_2_, Tb_2_O_3_, and Eu_2_O_3_ were weighed and ground completely for 30 min with an agate mortar (details of each doped sample can be found in Table S1 of the Supporting Information). Note that all of the doping percentages of lanthanide ions are atomic percentages in this work, and lanthanide ions were supposed to replace calcium ions. The mixture was transferred into an aluminum oxide crucible and sintered at 600 °C for 5 h. Then, the cooled mixture was ground again for 20 min using an agate mortar and heated to 1200 °C for 6 h. After cooling down to room temperature, the product was ground for another 10 min and collected for further use. All the sintering processes occurred under atmospheric conditions, and all heating and cooling rates were set to 5 °C/min.

### 3.2. Characterization

Powder X-ray diffraction patterns were recorded on a Bruker D8 Advance diffractometer with Cu Kα radiation as the incident beam. Scanning electron microscopy (SEM) measurements and the energy-dispersive X-ray spectroscopy (EDS) elemental analysis were conducted using a field emission scanning electron microscope (Thermo Fisher Scientific (Waltham, MA, USA) FIB-SEM GX4). X-ray photoelectron spectroscopy (XPS) analysis was carried out using an XPS Microprobe (Thermo SCIENTIFIC ESCALAB Xi+). The absorption spectra were collected with a UV-3600 UV-VIS-NIR spectrophotometer from Shimadzu (Kyoto, Japan). The excitation and emission spectra were measured by means of a Hitachi (Chiyoda City, Japan) F-7000 fluorescence spectrophotometer. The decay curves were obtained using an Edinburgh Instruments (Livingston, UK) FLS920 fluorescent spectrometer with a μs flash lamp as the excitation source.

### 3.3. Methods to Establish the Luminescent Patterns for Anti-Counterfeiting and Optical Information Encryption

Firstly, 2 g of as-prepared undoped CaZrO_3_, CaZrO_3_:8%Tb^3+^, CaZrO_3_:6%Eu^3+^, or CaZrO_3_:4%Tb^3+^/0.5%Eu^3+^ was mixed with 5 g of PVA aqueous solution, then underwent ultrasonic treatment to form a slurry. Then, the slurry was selectively coated on the patterned PMMA plate by dripping before being dried at 70 °C for 1 h. Photographs of the luminescent patterns were taken with a smartphone under 254 or 302 nm UV light in a dark box.

## 4. Conclusions

In summary, we synthesized undoped CaZrO_3_ with purple-blue luminescence and introduced green and red luminescence by doping lanthanide ions Tb^3+^ and Eu^3+^, respectively. In-depth research revealed the distinctly different excitation energy paths for luminescence from host CaZrO_3_ and dopants Tb^3+^ and Eu^3+^. This allowed the optimal excitation wavelengths to move away from each other to achieve luminescence in host CaZrO_3_ and dopants Tb^3+^ and Eu^3+^, which allowed us elaborative control of the luminescent color by modulating excitation wavelengths only in composition-fixed CaZrO_3_:Tb^3+^, CaZrO_3_:Eu^3+^ and CaZrO_3_:Tb^3+^/Eu^3+^. Thereafter, we demonstrated preliminary cases of applications for optical anti-counterfeiting and information encryption using this group of materials and common UV lamps. This study not only offers a generally applicable design strategy for multicolor luminescent regulation without the need to change the composition of the material, but will also help to develop advanced luminescent multi-functional rare-earth materials.

## Data Availability

Data are contained within the article and Supplementary Materials.

## Supplementary Materials

The following supporting information can be downloaded at https://www.mdpi.com/article/10.3390/molecules28227623/s1: Figure S1: Powder XRD patterns; Figure S2: Absorption spectrum and plot of (*αhν*)^2^ versus photo energy of CaZrO_3_; Figure S3: Emission spectra of CaZrO_3_:Tb^3+^ under 312 nm excitation, photographs of CaZrO_3_:Tb^3+^ under 245 nm excitation; Figure S4: Excitation spectra of CaZrO_3_:Eu^3+^ (2–8%), photographs of CaZrO_3_:Eu^3+^ (0–8%) under 280 nm excitation; Figure S5: Dependences of Log (*I*/*x*) against Log (*x*) for CaZrO_3_:Tb^3+^ (2–8%) and CaZrO_3_:Eu^3+^ (2–8%); Figure S6: Excitation spectra of CaZrO_3_:4%Tb^3+^/xEu^3+^ (x = 0.5–6%); Figure S7: Emission spectra of CaZrO_3_:Tb^3+^, CaZrO_3_:Eu^3+^ and CaZrO_3_:4%Tb^3+^/xEu^3+^ under 312 nm excitation; Figure S8: Excitation spectra, emission spectra, emission intensity variation, and luminescent photographs of CaZrO_3_:6%Eu^3+^; Table S1: Products and the detailed dosages of precursors for their synthesis; Table S2: Rietveld refinement results.
