# Supplementary material for: Excitation-Controlled Host–Guest Multicolor Luminescence in Lanthanide-Doped Calcium Zirconate for Information Encryption
Source: Molecules. 2023 Nov 16;28(22):7623. doi: 10.3390/molecules28227623 (PMC10675260; doi:10.3390/molecules28227623)
Supplement: Supplementary file 1 [file molecules-28-07623-s001.zip › molecules-2668199-supplementary.pdf]

Supporting Information

# Excitation-Controlled Host–Guest Multicolor Luminescence in Lanthanide-Doped Calcium Zirconate for Information Encryption

**Yangbo Wang [1,*], Yingdong Han [2], Runfa Liu [1], Cunping Duan [1] and Huaiyong Li [1,*]**

[1] School of Materials Science and Engineering, Liaocheng University, Liaocheng 252059, China
[2] College of Science, Civil Aviation University of China, Tianjin 300300, China; hansuo@126.com
* Correspondence: wangyangbo@lcu.edu.cn (Y.W.); lihuaiyong@lcu.edu.cn (H.L.)

**Table S1.** Products and the detailed dosage of precursors for their synthesis.

| Products | Dosage of precursors (mmol) | | | |
|---|---|---|---|---|
| | $CaCO_3$ | $ZrO_2$ | $Tb_2O_3$ | $Eu_2O_3$ |
| $CaZrO_3$ | 5 | 5 | — | — |
| $CaZrO_3$:2%$Tb^{3+}$ | 4.9 | 5 | 0.05 | — |
| $CaZrO_3$:4%$Tb^{3+}$ | 4.8 | 5 | 0.1 | — |
| $CaZrO_3$:6%$Tb^{3+}$ | 4.7 | 5 | 0.15 | — |
| $CaZrO_3$:8%$Tb^{3+}$ | 4.6 | 5 | 0.2 | — |
| $CaZrO_3$:2%$Eu^{3+}$ | 4.9 | 5 | — | 0.05 |
| $CaZrO_3$:4%$Eu^{3+}$ | 4.8 | 5 | — | 0.1 |
| $CaZrO_3$:6%$Eu^{3+}$ | 4.7 | 5 | — | 0.15 |
| $CaZrO_3$:8%$Eu^{3+}$ | 4.6 | 5 | — | 0.2 |
| $CaZrO_3$:4%$Tb^{3+}$/0.5%$Eu^{3+}$ | 4.775 | 5 | 0.1 | 0.0125 |
| $CaZrO_3$:4%$Tb^{3+}$/1%$Eu^{3+}$ | 4.75 | 5 | 0.1 | 0.025 |
| $CaZrO_3$:4%$Tb^{3+}$/2%$Eu^{3+}$ | 4.7 | 5 | 0.1 | 0.05 |
| $CaZrO_3$:4%$Tb^{3+}$/4%$Eu^{3+}$ | 4.6 | 5 | 0.1 | 0.1 |
| $CaZrO_3$:4%$Tb^{3+}$/6%$Eu^{3+}$ | 4.5 | 5 | 0.1 | 0.15 |

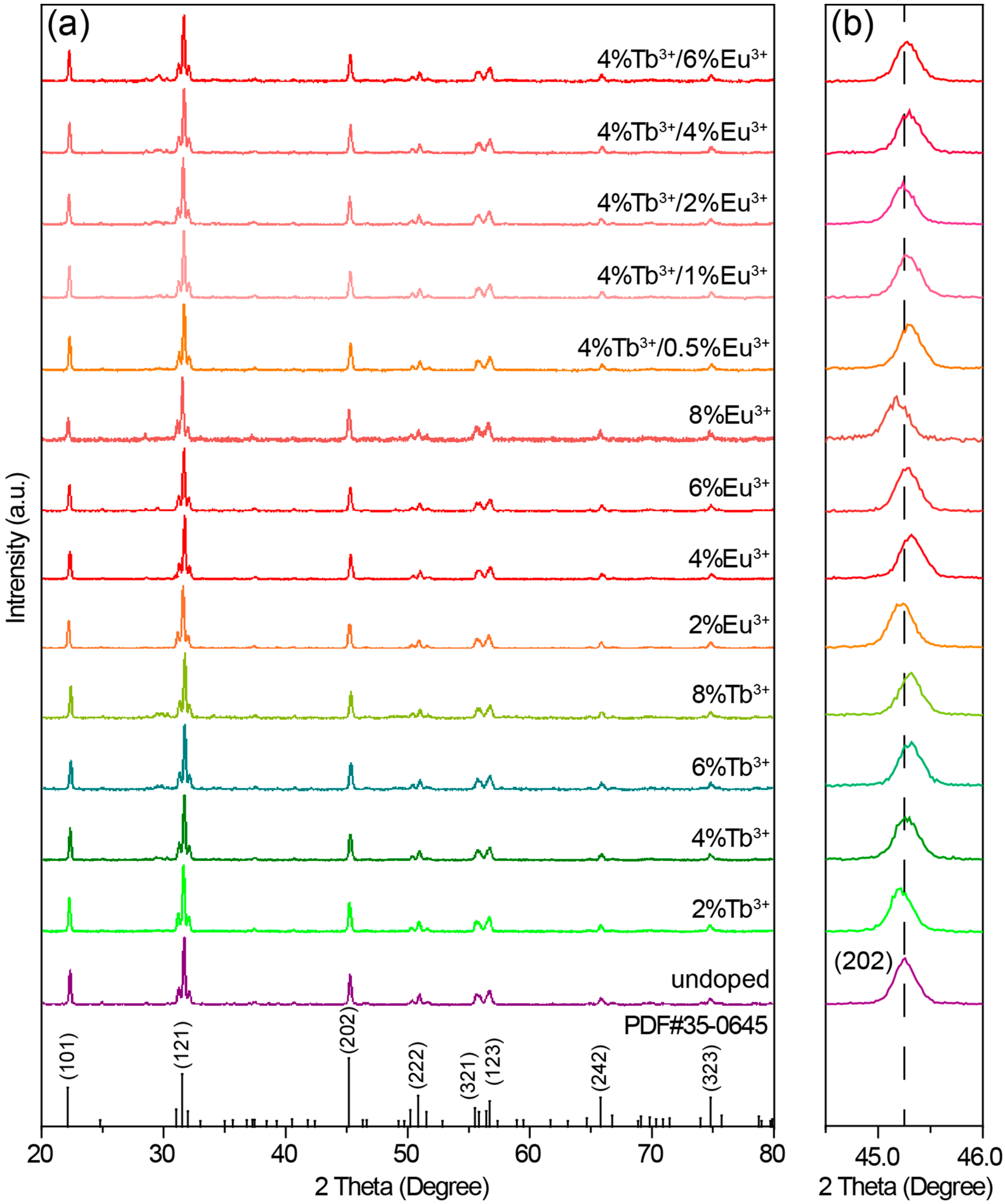


**Figure S1.** (a) Powder XRD patterns of undoped $CaZrO_3$, $CaZrO_3$:$Tb^{3+}$ (2–8%), $CaZrO_3$:$Eu^{3+}$ (2–8%), and $CaZrO_3$:4%$Tb^{3+}$/x$Eu^{3+}$ (x = 0.5–6%). The bars at the bottom are the reference standard patterns of $CaZrO_3$ (PDF card No. 35–0645). (b) The diffraction peaks of (202) planes of corresponding samples.

**Table S2.** Data of Rietveld refinement for undoped $CaZrO_3$ and $CaZrO_3$:4%$Tb^{3+}$0.5%$Eu^{3+}$.

| Parameter | $CaZrO_3$ | $CaZrO_3$:4%$Tb^{3+}$0.5%$Eu^{3+}$ |
|---|---|---|
| Space group | *Pnma* | *Pnma* |
| $a$ (Å) | 5.6008 | 5.5995 |
| $b$ (Å) | 8.0205 | 8.0194 |
| $c$ (Å) | 5.7520 | 5.7474 |
| $\alpha=\beta=\gamma$ (°) | 90 | 90 |
| $V$ (Å$^3$) | 258.39 | 258.08 |
| $R_{wp}$ (%) | 13.55 | 13.96 |
| $R_p$ (%) | 9.95 | 10.30 |
| $\chi^2$ | 5.6 | 6.6 |

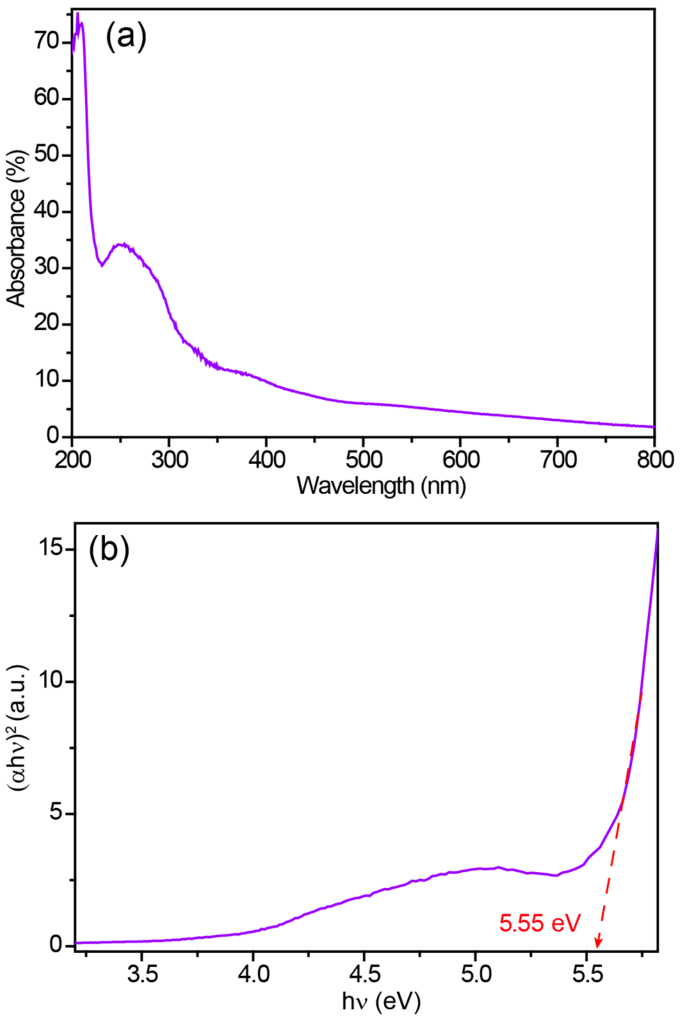


**Figure S2.** (a) Absorption spectrum and (b) plot of $(\alpha h\nu)^2$ versus photo energy ($h\nu$) of $CaZrO_3$.

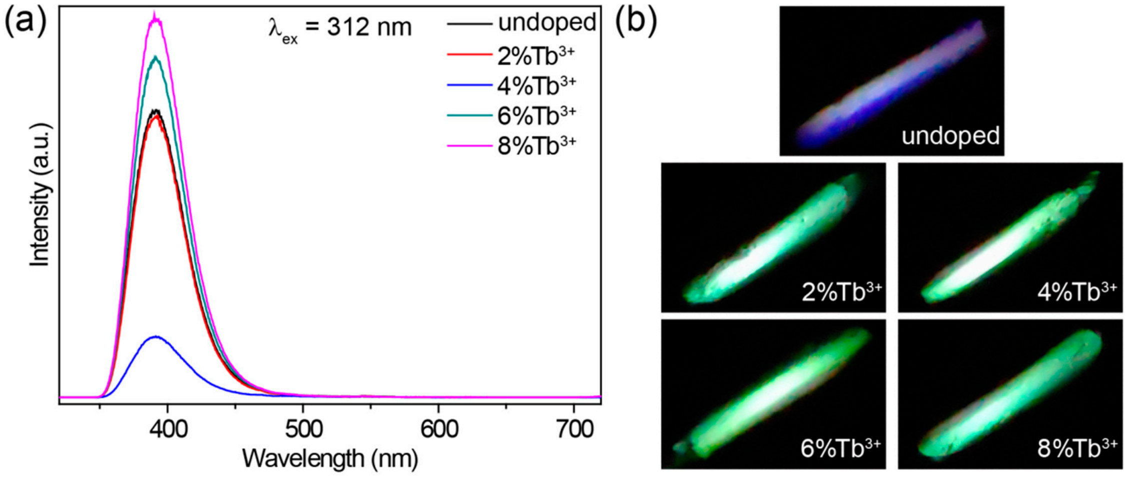


**Figure S3.** (a) Emission spectra of $CaZrO_3$:$Tb^{3+}$ (0–8%) under 312 nm excitation. (b) Digital photographs of $CaZrO_3$:$Tb^{3+}$ (0–8%) under 245 nm excitation.

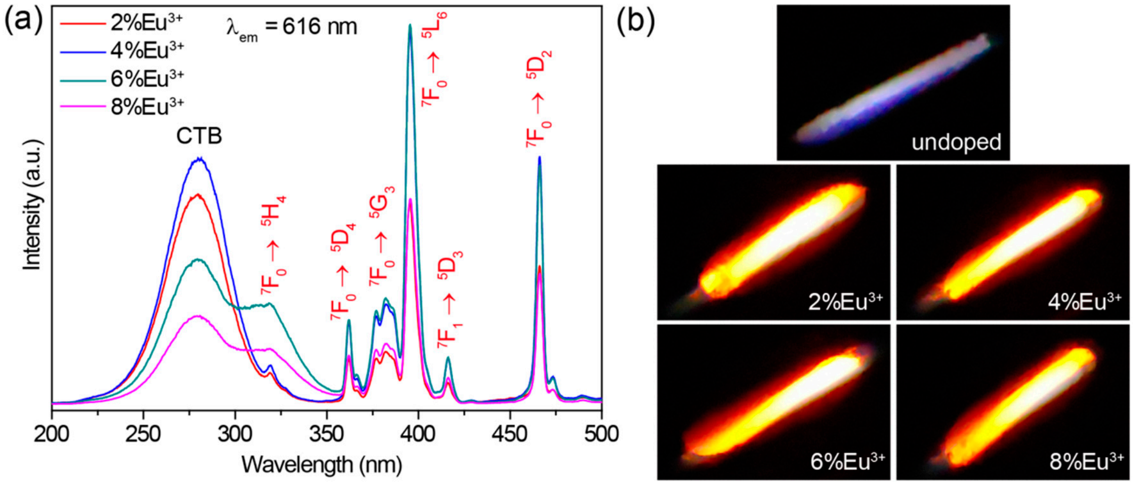


**Figure S4.** (a) Excitation spectra of $CaZrO_3:Eu^{3+}$ (2–8%) by monitoring emissions at 616 nm. (b) Digital photographs of $CaZrO_3:Eu^{3+}$ (0–8%) under 280 nm excitation.

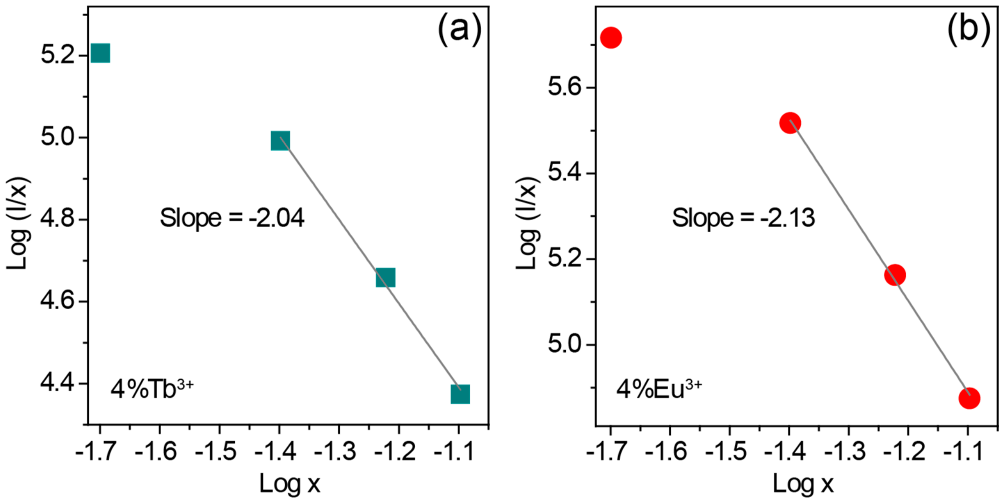


**Figure S5.** Dependences of Log ($I/x$) against Log ($x$) for (a) $CaZrO_3:Tb^{3+}$ (2–8%) and (b) $CaZrO_3:Eu^{3+}$ (2–8%).

The mode of the multipole-multipole interaction was identified by using the following equation:

$$I/x = k/(1 + \beta x^{\theta/3})^{-1}$$

where $I$ is the integrated luminescence intensity, $x$ is the doping concentration, $\beta$ and $k$ are constants, $\theta$ is an index corresponding to the multipole-multipole interaction mode. The electric dipole-dipole, dipole-quadrupole, and quadrupole-quadrupole interaction can be identified when the determined value of $\theta$ is about 6, 8, and 10, respectively. We plotted log ($I/x$) as a function of log ($x$) and fitted the results exceeding the critical doping concentration to a straight line as present in Fig. S5. Then the values of $\theta$ were obtained from the slopes ($-\theta/3$) as 6.12 and 6.39. So, the mode of the multipole-multipole interaction is the dipole-dipole interaction.

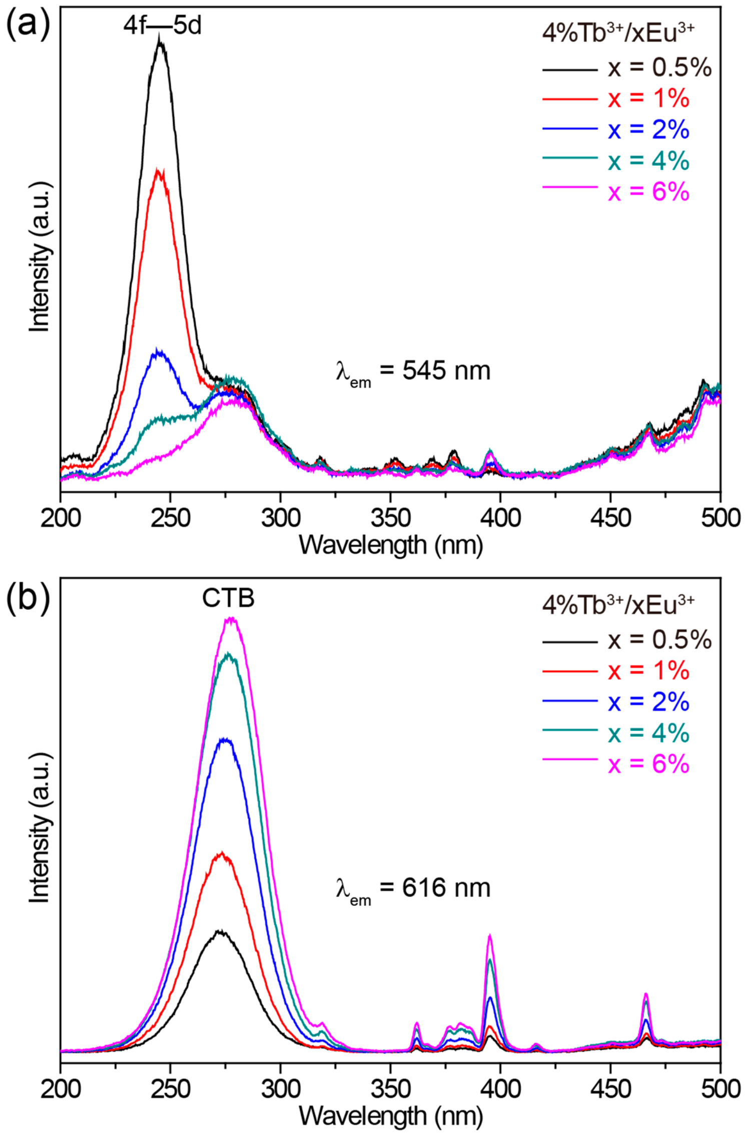


**Figure S6.** Excitation spectra of $CaZrO_3$:4%$Tb^{3+}$/x$Eu^{3+}$ (x = 0.5–6%) by monitoring emissions at (a) 545 and (b) 616 nm.

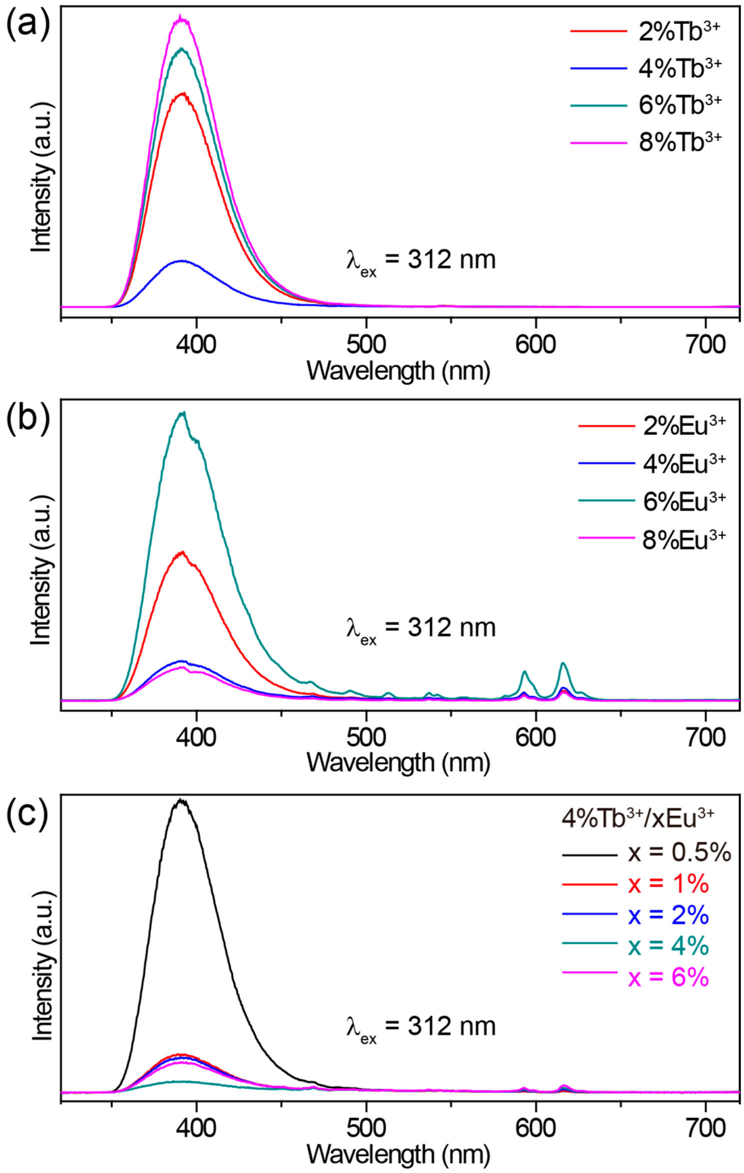


**Figure S7.** Emission spectra of (a) $CaZrO_3:Tb^{3+}$ (2–8%), (b) $CaZrO_3:Eu^{3+}$ (2–8%), and (c) $CaZrO_3$:4%$Tb^{3+}$/x$Eu^{3+}$ (x = 0.5–6%) under 312 nm excitation.

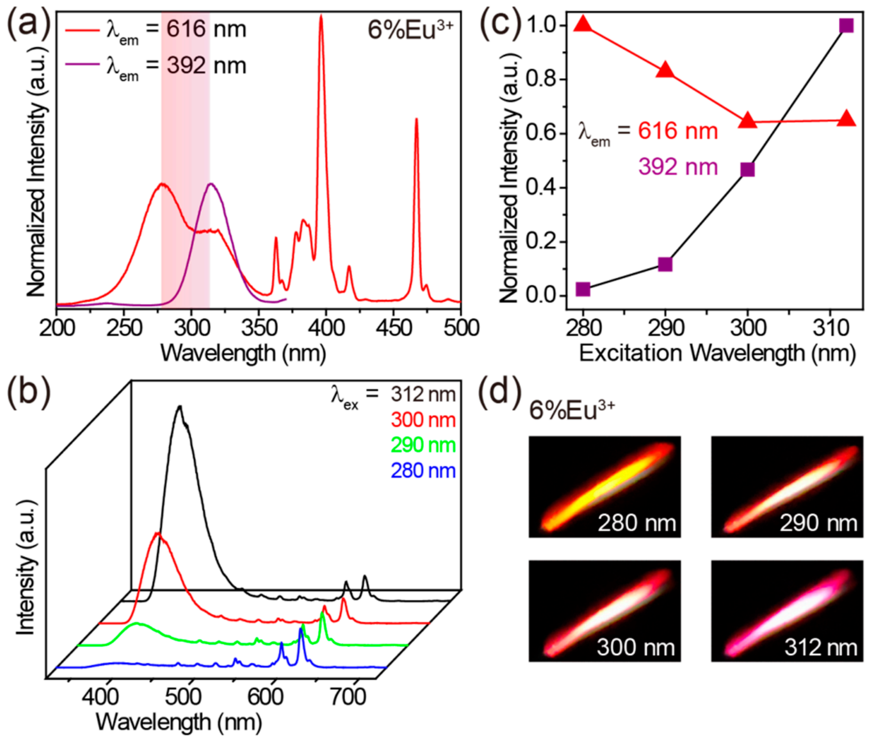


**Figure S8.** (a) Excitation spectra of $CaZrO_3$:6%$Eu^{3+}$ by monitoring emissions at 616 and 392 nm. (b) Emission spectra and (c) intensity variation of $CaZrO_3$:6%$Eu^{3+}$ under 280–312 nm excitations. Digital photographs of $CaZrO_3$:6%$Eu^{3+}$ under different excitation wavelengths.
